# Comparison of three occlusal splint material systems manufactured by conventional, subtractive, and additive techniques

**DOI:** 10.1007/s00784-026-07250-7

**Published:** 2026-11-09

**Authors:** Mariana Farias Rocha, Isabela Vitória de Oliveira Nunes, Natasha Rohrig Assumpção da Motta, Anna Júlia Mendonça Paraíso, May Anny Alves Fraga, Cleocir José Dalmaschio, Rafael Ratto de Moraes, Nathalia Silveira Finck, Juliana Malacarne-Zanon

**Affiliations:** 1 School of Dentistry, Federal University of Espírito Santo (UFES), Vitória, ES Brazil; 2 Centro Universitário FAESA, Vitória, ES Brazil; 3 Department of Restorative Dentistry, Endodontics and Dental Materials, Bauru School of Dentistry, University of São Paulo (FOB-USP), Bauru, SP Brazil; 4 Department of Chemistry, Federal University of Espírito Santo (UFES), Vitória, ES Brazil; 5 Department of Restorative Dentistry, Federal University of Pelotas (UFPel), Pelotas, RS Brazil; 6 Department of Prosthodontics, Federal University of Espírito Santo (UFES), Vitória, ES Brazil

**Keywords:** Occlusal splints, Polymethyl methacrylate, CAD/CAM, 3D printing, Dynamic mechanical analysis, Water sorption

## Abstract

**Objectives:**

To compare the thermal–viscoelastic behavior, hygroscopic properties, color stability, and surface morphology of three commercial occlusal splint material systems produced by conventional heat polymerization, CAD/CAM milling, and 3D printing. The null hypothesis was that the three material systems would not differ in thermal-viscoelastic behavior, hygroscopic properties, or color stability. Surface morphology was assessed qualitatively as a complementary outcome.

**Materials and methods:**

Heat-polymerized polymethyl methacrylate (HEC), CAD/CAM-milled polymethyl methacrylate (CAD-M), and a 3D-printed photopolymer resin (3DP) were evaluated. Dynamic mechanical analysis was performed to determine glass transition temperature (Tg), tan δ, and thermal heterogeneity (*n* = 5). Water sorption (WS), solubility (SL), and color change after coffee immersion (ΔE00) were assessed (*n* = 8). Surface morphology was qualitatively analyzed by atomic force microscopy and scanning electron microscopy.

**Results:**

Significant differences were observed among the material systems for thermal and hygroscopic outcomes. CAD-M presented the highest mean Tg (149.58 ± 0.95 °C), the lowest water sorption (18.76 ± 0.56 µg/mm³), the lowest solubility (2.59 ± 0.48 µg/mm³), and a ΔE00 of 2.29 ± 1.20 after 7 days of coffee immersion. Conversely, 3DP showed the lowest Tg (83.18 ± 7.87 °C), highest WS (35.12 ± 0.82 µg/mm³), highest SL (6.16 ± 0.40 µg/mm³), greatest color change, and a more irregular surface morphology.

**Conclusions:**

The three evaluated commercial material systems differed in thermal-viscoelastic behavior, hygroscopic properties, color stability, and surface morphology. Among the specific systems tested, CAD-M showed the most favorable overall laboratory profile. Because each manufacturing approach was represented by a single commercial material with a distinct composition, these differences cannot be attributed exclusively to the manufacturing route.

**Clinical relevance:**

Under the in vitro conditions tested, the milled material system showed a higher glass transition temperature and lower water sorption, solubility and coffee-induced discoloration than the 3D-printed system. Whether these laboratory differences translate into differences in the clinical performance of occlusal splints was not assessed in this study.

**Supplementary Information:**

The online version contains supplementary material available at https://doi.org/10.1007/s00784-026-07250-7.

## Introduction

Occlusal splints are widely prescribed for the management of bruxism and temporomandibular disorders (TMDs). Nevertheless, much of the available evidence on acrylic-based materials has traditionally focused on interim restorations and denture base resins rather than on splint-specific performance, leaving key aspects of long-term behavior insufficiently characterized [1–4]. During clinical use, splints are repeatedly exposed to mechanical loading, intraoral temperature fluctuations, and a humid environment. These conditions may promote water uptake and the leaching of soluble components, which can alter polymer mobility and, consequently, affect stiffness, dimensional stability, and durability over time [5, 6]. In addition, splints are routinely challenged by dietary chromogens, and surface characteristics may influence pigment absorption and penetration, contributing to esthetic degradation.

Dynamic mechanical analysis (DMA) enables the evaluation of temperature-dependent viscoelastic parameters, such as the storage modulus (E′), loss modulus (E″), tan δ, and glass transition temperature (Tg), which are sensitive to polymer network integrity and molecular mobility. However, studies that integrate DMA with hygroscopic testing and surface characterization remain limited for the main manufacturing routes currently used for occlusal splints: conventional heat-polymerized PMMA (HEC), CAD/CAM-milled PMMA (CAD-M), and additively manufactured 3D-printed photopolymer resins (3DP) [7–13]. Moreover, the relationships between hygroscopic behavior, staining susceptibility (color change), and surface morphology assessed by atomic force microscopy (AFM) and scanning electron microscopy (SEM) have not been consistently addressed in a unified experimental design.

Therefore, this study aimed to compare HEC, CAD-M, and 3DP materials used for occlusal splints by jointly evaluating their thermal and viscoelastic behavior using DMA, water sorption and solubility, color stability, and surface morphology by AFM and SEM. By comparing three commercial material systems representing conventional, subtractive, and additive manufacturing approaches, this study sought to characterize differences in thermal-viscoelastic behavior, water-related properties, color stability, and surface morphology.

The null hypothesis was that the three evaluated material systems would not differ in thermal-viscoelastic behavior, hygroscopic properties, or color stability. Surface morphology was assessed qualitatively as a complementary outcome.

## Materials and methods

### Experimental design and groups

Three commercial material systems used for the fabrication of occlusal splints were selected to represent three clinically relevant manufacturing workflows: conventional heat polymerization (HEC), subtractive CAD/CAM milling (CAD-M), and additive 3D printing (3DP). The purpose of the material selection was to compare representative material-processing systems rather than to isolate manufacturing route as an independent factor. Because each workflow was represented by a different commercial material, differences in chemical composition were considered a potential contributing factor to the observed outcomes. Disk-shaped samples (Ø 15 mm × 1 mm) were prepared for water sorption/solubility and color change tests (*n* = 8 per group). Rectangular bars (2.0 × 6.0 × 30.0 mm) were prepared for dynamic mechanical analysis (DMA) (*n* = 5 per group). The trade name, manufacturer, batch number, and composition are presented in Table 1. All the specimen geometries were designed using Exocad software (Darmstadt, Germany).

**Table 1:** Materials evaluated in the study, including commercial name, manufacturer, lot number, and composition

Trade name	Manufacturer	Lot number	Composition/notes^a^
Smart Print Bio Bite Splint	Smart Dent, São Carlos, SP, Brazil.	PBSF051/24	Monomers, Oligomers, Photoinitiator, Pigments, Stabilizers.
Mult Block	Mult Produtos Odontológicos, Pirassununga, SP, Brazil.	0125	Polymethyl methacrylate, EDMA, Fluorescent, organic additives, and biocompatible pigments.
VIPI CRIL PLUS	Vipi Produtos Odontológicos, Pirassununga, SP, Brazil.	0000000113	Powder (Polymethyl methacrylate, Polypropylene, Pigments) Liquid (Methyl methacrylate EDMA -Crosslink; Inhibitor)

^a^Information provided by the manufacturers

### 3D printing and postprocessing (3DP)

STL files were printed using a Mars 4 Ultra printer (Elegoo, Shenzhen, China) with a 50 μm layer thickness. Disk samples were printed at a 45° build angle and bar samples at a 150° build angle, following a previously described orientation protocol [14]. Postprocessing consisted of isopropyl alcohol rinsing (3 min) and UV postcuring using an Anycubic Wash and Cure unit (Shenzhen Anycubic Technology Co., Shenzhen, China; 405/365 nm) according to the manufacturer’s instructions. The specimens were positioned at the periphery of the chamber, and the total post-curing time was 10 min, equally distributed between both surfaces. Supports were manually removed, and the samples were finished with 400-, 600-, 1200-, and 2000-grit SiC papers (Norton Abrasives, Guarulhos, SP, Brazil) [15].

### CAD/CAM milling (CAD-M)

The samples were milled from a PMMA disk (Mult Block; Mult Produtos Odontológicos) using an X-mill 500 Plus unit (XTCERA). All surfaces were finished with 400-, 600-, 1200, and 2000-grit SiC papers (Norton Abrasivos, Guarulhos, SP, Brazil).

### Conventional heat polymerization (HEC)

To standardize the specimen geometry across groups, burnout-compatible sacrificial patterns corresponding to the final specimen dimensions were 3D-printed in (Cosmos Castable DLP photopolymer resin, Yller Digital, Pelotas, RS, Brazil) and used as templates for mold production. This resin was used solely to fabricate the sacrificial patterns and was not evaluated as an experimental material.

The patterns were cleaned, the supports were removed, and the surfaces were finished to ensure dimensional fidelity. Each pattern was invested and eliminated by burnout to obtain molds. HEC samples were fabricated with VIPI acrylic resin (Vipi Produtos Odontológicos, Pirassununga, SP, Brazil) using compression molding. Polymerization was performed in a water bath at 85–90 °C for 8 h using a hydraulic press (Delta, Vipi-Delta Máquinas Especiais). The samples were then finished with 400-, 600-, 1200-, and 2000-grit SiC papers to match the CAD-M and 3DP finishing protocol.

### Dynamic mechanical analysis (DMA)

**Fig. 1:**
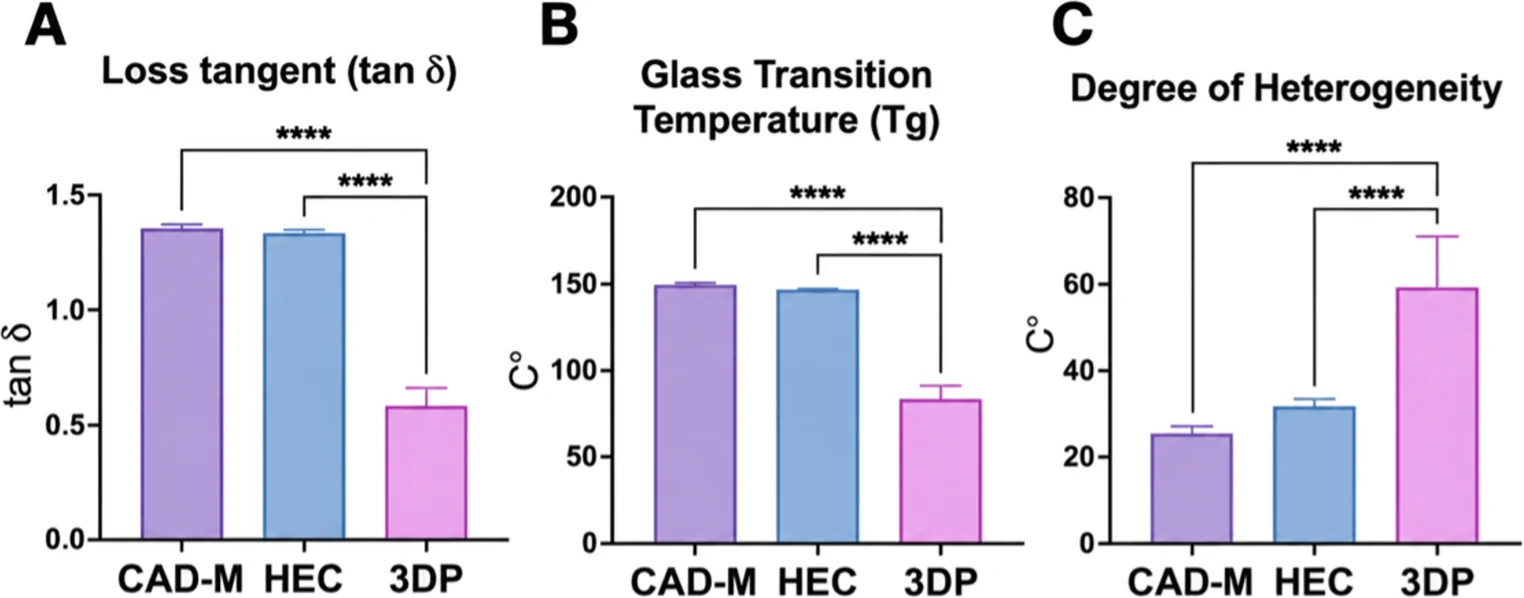
Mean ± standard deviation of (**A**) tan δ (dimensionless), (**B**) glass transition temperature (Tg, °C) determined from the tan δ peak temperature, and (**C**) thermal heterogeneity expressed as the tan δ peak width at half maximum (FWHM, °C) for HEC, CAD-M, and 3DP at 10 Hz

Dynamic mechanical analysis (DMA) was performed on rectangular specimens (*n* = 5) using a DMA 8000 instrument (PerkinElmer, Waltham, MA, USA) operating in single-cantilever mode [16, 17]. The analyses were conducted under air atmosphere from − 20 °C to 200 °C at a heating rate of 5 °C/min, with an oscillation amplitude of 0.05 mm and frequencies of 1 and 10 Hz. The glass transition temperature (Tg) was determined from the maximum of the tan δ curve, whereas thermal heterogeneity was quantified as the full width at half maximum (FWHM, °C) of the tan δ peak. Although measurements were acquired at 1 and 10 Hz, the parameters reported in Fig. 1 and used for statistical comparisons were derived from the 10-Hz curves.

### Water sorption and solubility (ISO 4049)

Water sorption and solubility were evaluated according to ISO 4049 [18] using disk samples (*n* = 8). The samples were desiccated at 37 °C to constant mass (m₁), immersed in distilled water at 37 °C for 7 days to obtain saturated mass (m₂), and redesiccated to a constant mass (m₃). Water sorption (WS) and solubility (SL) were calculated as WS = (m₂ − m₃)/V and SL = (m₁ − m₃)/V, where V is the sample volume.

### Color measurement and coffee staining

Color measurements were conducted at the central point of each specimen against a standardized gray background using a VITA Easyshade V spectrophotometer (VITA Zahnfabrik, Bad Säckingen, Germany), with device calibration performed before each session. Color coordinates were recorded at baseline, i.e., after 24 h immersion in distilled water at 37 °C, and after immersion in a freshly prepared Nescafé^®^ instant coffee solution (2 g/100 mL) at 37 °C for 3 and 7 days, with daily renewal of the solution. After each period, the samples were rinsed, gently air-dried, and remeasured under the same conditions. Three consecutive readings per sample were averaged, and the ΔE_00_ was calculated using the CIEDE2000 formula (ΔE_00_).

### Surface topography and microstructural analysis (AFM and SEM)

**Fig. 2:**
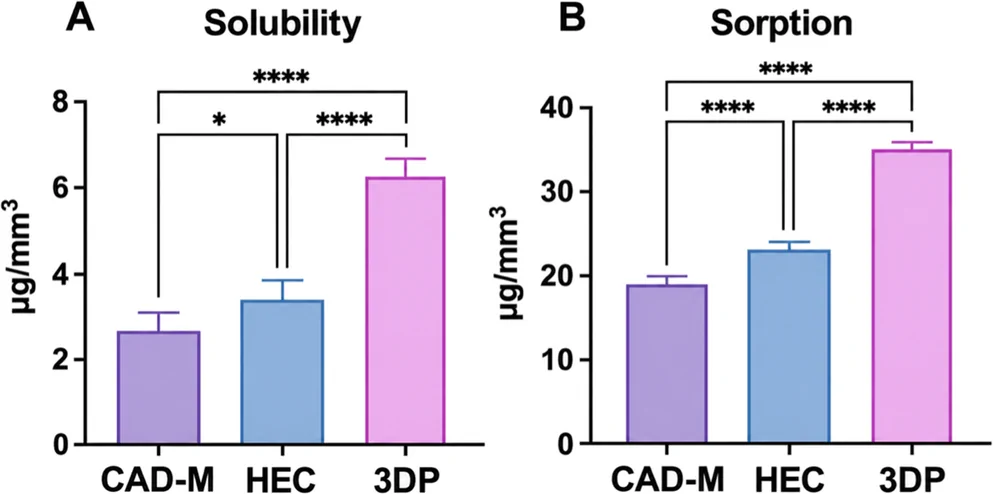
Mean ± standard deviation (µg/mm³) of water solubility (**A**) and sorption (**B**) for CAD-M, HEC, and 3DP. Asterisks indicate statistically significant differences according to Tukey’s multiple-comparison test (α = 0.05). Sample sizes were n = 8 for HEC, CAD-M and 3DP

Nanoscale surface topography was examined by atomic force microscopy (AFM) in tapping mode using a WITec alpha300 R instrument (WITec GmbH, Ulm, Germany). All specimens were scanned over matched 15 × 15 μm areas (*n* = 3 per material). Height scales are indicated individually in Fig. 2, with z-ranges of 2.6, 1.3, and 1.5 μm for CAD-M, HEC, and 3DP, respectively. Because AFM was used for qualitative visualization of post-immersion nanoscale surface features, the images were interpreted descriptively and were not used for quantitative roughness comparisons among groups.

Additional samples were characterised using a field-emission scanning electron microscope (FEG-SEM, Apreo 2 S, Thermo Fisher Scientific, The Netherlands) operated at an accelerating voltage of up to 5 kV, using a secondary electron detector. The samples were sputter-coated with gold prior to imaging. Micrographs were acquired from standardized surface areas at standardized magnifications (×500 and ×2000). The SEM allows an accelerating voltage range of 0.3–30 kV.

### Data analysis

Sample sizes were defined based on previous studies employing comparable methodologies for polymeric dental materials [10, 15], and dynamic mechanical analysis was performed according to established standards [16, 17]. Normality and homoscedasticity were assessed using the Shapiro–Wilk and Levene tests, respectively. Water sorption, solubility, tan δ, glass transition temperature (Tg), and degree of heterogeneity were analyzed using one-way ANOVA followed by Tukey’s post hoc test (α = 0.05). Effect sizes were expressed as eta squared (η²). Color change (ΔE00) was analyzed using two-way ANOVA considering material and time as factors, followed by Tukey’s multiple-comparison test. Effect sizes for the two-way ANOVA were expressed as partial eta squared (ηp²). L*, a*, and b* coordinates were presented descriptively. Statistical analyses were performed using GraphPad Prism 10.2.3 (GraphPad Software, Boston, MA, USA), with a significance level of 5%.

## Results

### Dynamic mechanical properties

Significant differences among material systems were observed for tan δ, Tg, and degree of heterogeneity. For tan δ, one-way ANOVA showed a significant material effect (F(2,12) = 446.198, *p* = 5.457 × 10⁻¹², η² = 0.987). HEC (1.337 ± 0.014) and CAD-M (1.358 ± 0.017) did not differ significantly from each other (adjusted *p* = 0.773976), whereas both presented significantly higher values than 3DP (HEC vs. 3DP: adjusted *p* = 1.397 × 10⁻¹¹; CAD-M vs. 3DP: adjusted *p* = 9.333 × 10⁻¹²).

Tg also differed significantly among materials (F(2,12) = 336.240, *p* = 2.904 × 10⁻¹¹, η² = 0.982). CAD-M (149.58 ± 0.95 °C) and HEC (146.86 ± 0.42 °C) did not differ significantly (adjusted *p* = 0.627419), while both exhibited significantly higher Tg values than 3DP (HEC vs. 3DP: adjusted *p* = 1.304 × 10⁻¹⁰; CAD-M vs. 3DP: adjusted *p* = 7.207 × 10⁻¹¹).

Degree of heterogeneity, expressed as FWHM, also differed among groups (F(2,12) = 34.490, *p* = 1.059 × 10⁻⁵, η² = 0.852). HEC (31.77 ± 1.70 °C) and CAD-M (25.52 ± 1.70 °C) did not differ significantly (adjusted *p* = 0.352347), whereas 3DP showed significantly greater heterogeneity than both HEC (adjusted *p* = 9.861 × 10⁻⁵) and CAD-M (adjusted *p* = 1.343 × 10⁻⁵).

The individual DMA curves for all specimens at 10 Hz are provided in Supplementary Figure S1.

### Water sorption and solubility

One-way ANOVA indicated significant differences among the material systems for both water sorption and solubility. Water sorption was highest for 3DP (35.124 ± 0.821 µg/mm³), followed by HEC (23.016 ± 0.619 µg/mm³), and lowest for CAD-M (18.761 ± 0.561 µg/mm³) (F(2,21) = 1260.9, *p* < 0.001, η² = 0.992).

Solubility also differed significantly among the material systems (F(2,21) = 130.6, *p* < 0.001, η² = 0.926). The highest solubility was observed for 3DP (6.157 ± 0.398 µg/mm³), followed by HEC (3.328 ± 0.510 µg/mm³), whereas CAD-M presented the lowest value (2.585 ± 0.484 µg/mm³). Tukey’s post hoc test indicated significant differences among the three material systems (*p* < 0.05).

### Color change

**Fig. 3:**
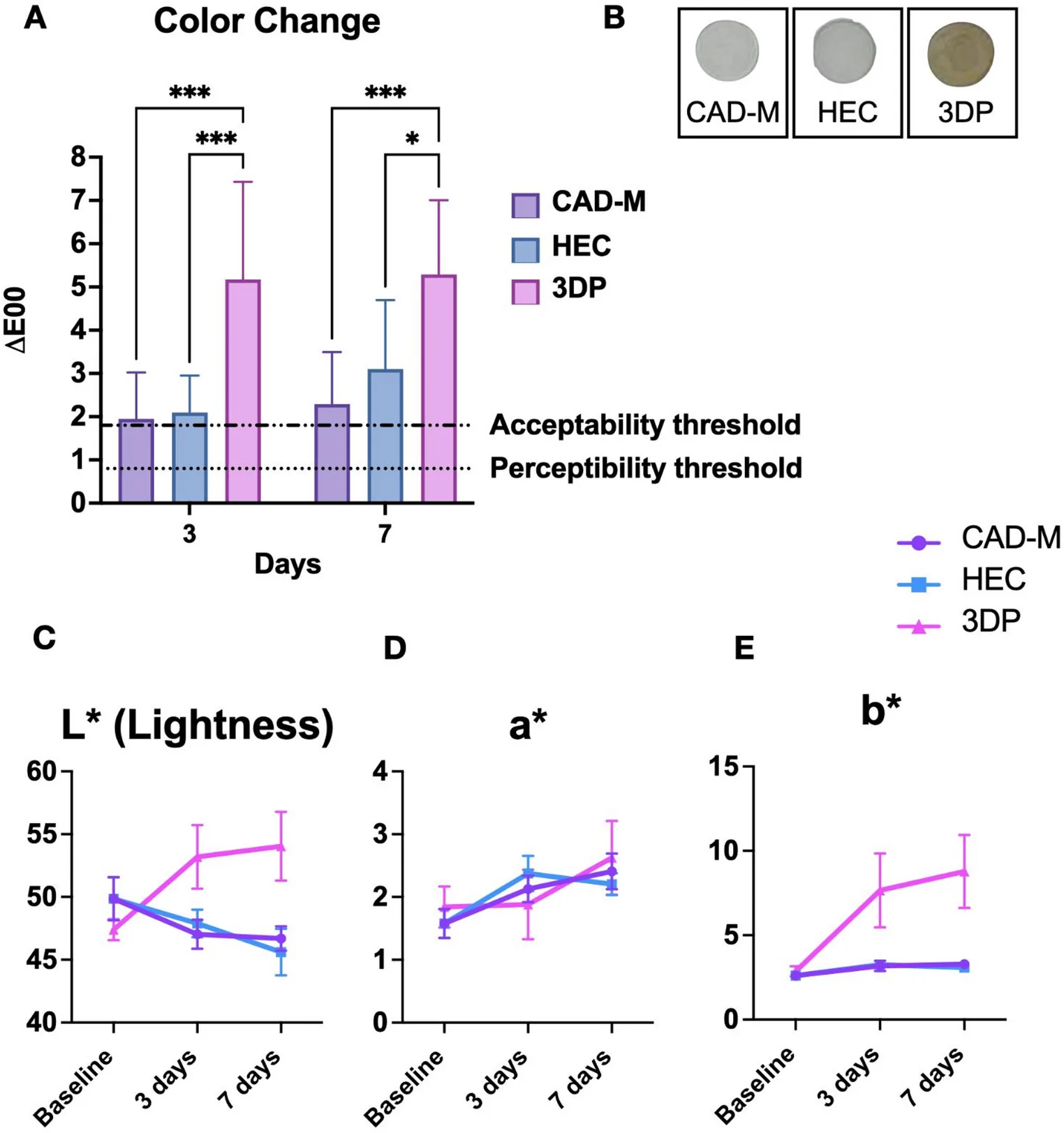
(**A**) Mean ± standard deviation of ΔE00 after coffee immersion for HEC, CAD-M, and 3DP at 3 and 7 days (*n* = 8 per material). (**B**) Representative images of the specimens after coffee immersion. (**C**) Lightness (L*), (**D**) a* (green–red axis), and (**E**) b* (blue–yellow axis) values at baseline, 3 days, and 7 days (*n* = 5 per material). ΔE00 data were analyzed by two-way ANOVA followed by Tukey’s multiple-comparison test (α = 0.05). Panels **C** to **E** are presented descriptively

Two-way ANOVA revealed a significant effect of material on ΔE00 (F(2,42) = 19.397, *p* = 1.080 × 10⁻⁶, ηp² = 0.480), whereas the effect of time (F(1,42) = 1.239, *p* = 0.2719, ηp² = 0.029) and the material × time interaction (F(2,42) = 0.368, *p* = 0.6944, ηp² = 0.017) were not significant (Fig. 3).

At 3 days, ΔE00 values were 1.950 ± 1.074 for CAD-M, 2.096 ± 0.856 for HEC, and 5.174 ± 2.255 for 3DP. At 7 days, the corresponding values were 2.294 ± 1.200, 3.102 ± 1.590, and 5.291 ± 1.717, respectively. At 3 days, 3DP showed significantly greater discoloration than HEC (adjusted *p* = 0.000657) and CAD-M (adjusted *p* = 0.000362), whereas HEC and CAD-M did not differ significantly (adjusted *p* = 0.996484). At 7 days, 3DP also showed significantly greater discoloration than HEC (adjusted *p* = 0.018698) and CAD-M (adjusted *p* = 0.000906), while HEC and CAD-M remained statistically similar (adjusted *p* = 0.648544). No significant difference between 3 and 7 days was detected within HEC (adjusted *p* = 0.193452), CAD-M (adjusted *p* = 0.653221), or 3DP (adjusted *p* = 0.878253).

### Surface morphology analyses (AFM and SEM)

Representative AFM images obtained after the color-change protocol showed qualitative differences in surface appearance among the evaluated materials. The 3DP specimens exhibited visually more irregular topographic features, whereas CAD-M showed a comparatively more uniform appearance and HEC an intermediate pattern. Because AFM was used qualitatively, these observations were not interpreted as quantitative differences in surface roughness.

SEM micrographs acquired from standardized surface areas at ×500 and ×2000 further supported these morphological distinctions (Fig. 2). CAD-M exhibited machining-related linear features with minimal evidence of surface defects. In contrast, 3DP showed visible layer boundaries, depressions, and discontinuities characteristic of additive manufacturing. HEC displayed a relatively homogeneous surface with interglobular features and occasional microvoids, which is consistent with powder–liquid processing.

## Discussion

A comparative assessment of three commercial occlusal splint material systems, each produced by a different manufacturing approach, revealed marked differences in thermal-viscoelastic behavior, hygroscopic response, and surface morphology. For the DMA outcomes, CAD-M and HEC showed similar tan δ, Tg, and degree of heterogeneity, whereas 3DP differed significantly from both PMMA-based materials. CAD-M showed numerically higher Tg and lower heterogeneity than HEC, although these differences were not statistically significant. The higher Tg and lower heterogeneity observed for the PMMA-based materials compared with 3DP suggest reduced segmental mobility and a more uniform thermomechanical response. These findings are consistent with reports describing differences in mechanical behavior among milled, heat-polymerized, and additively manufactured splint materials [19, 20].

The 3DP samples exhibited the lowest tan δ, indicating lower energy dissipation relative to elastic storage and a less pronounced damping response. This behavior may be associated with increased molecular mobility and reduced network rigidity, particularly when considered alongside the lower Tg and structural heterogeneity observed for 3DP materials [15, 17], which may arise from a lower effective cross-link density and/or weaker interlayer cohesion inherent to layer-by-layer fabrication. In contrast, the HEC showed tan δ values comparable to CAD-M and significantly higher than 3DP. Consistent with this viscoelastic profile, 3DP also showed the highest heterogeneity and the lowest Tg (Fig. 1). Such a pattern suggests a more heterogeneous network with reduced thermal resistance, potentially attributable to incomplete photopolymerization, oxygen-inhibition effects, and interlaminar interfaces that may act as mechanically weaker zones [21, 22]. The lower glass transition temperature of 3DP indicates that its segmental relaxation occurs at a lower temperature than that of the other two systems. Because neither cyclic loading nor deformation was evaluated here, the clinical consequences of this difference cannot be inferred from the present data. In comparison, the HEC showed thermal parameters comparable to CAD-M and significantly different from 3DP. Although heat-polymerized PMMA may present limitations related to residual monomers and variability in conversion, it remains a clinically acceptable option in situations with lower mechanical demands, particularly given its accessibility and cost-effectiveness.

**Fig. 4:**
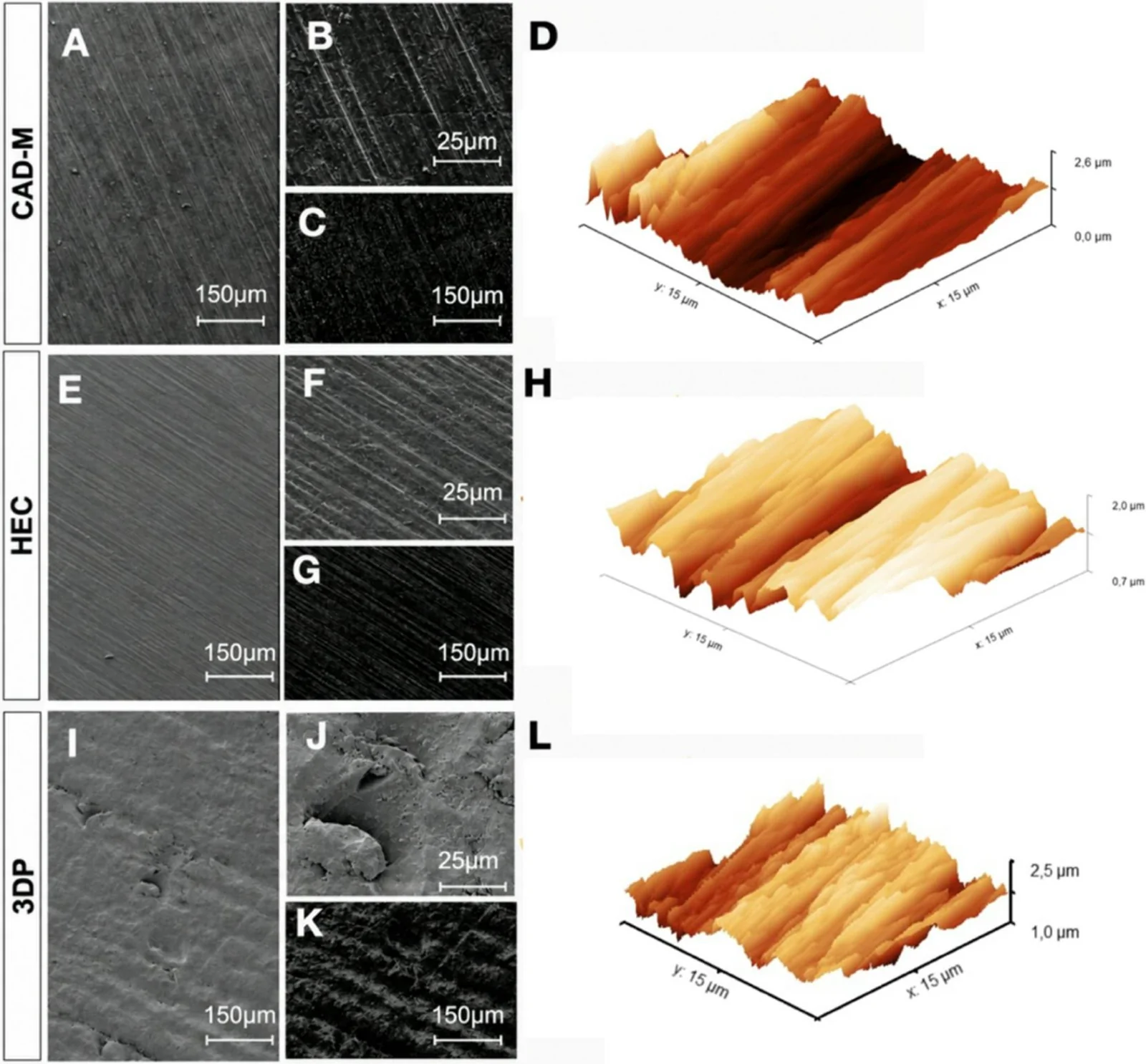
Representative surface morphology of HEC, CAD-M, and 3DP. SEM micrographs (FEG-SEM; Apreo 2 S, Thermo Fisher Scientific, Eindhoven, The Netherlands; gold-coated; x500 and ×2000; images acquired from standardized surface areas) illustrate microstructural surface features and manufacturing-related defects. (**A**, **E**, **I**) SEM micrographs at ×500 magnification. (**B**, **F**, **J**) SEM micrographs at ×2000 magnification. (**C**, **G**, and **K**) Edge mask overlaid on the original image in x500 to highlight structural boundaries (edges detected using the ImageJ – Edges tool). (**D**, **H**, and **L**) AFM images acquired in tapping mode provide qualitative nanoscale topography. AFM images were acquired over matched 15 × 15 μm areas; height scales are indicated individually in each panel. The images were interpreted qualitatively and were not used for quantitative roughness comparisons

For the hygroscopic outcomes, CAD-M presented the lowest water sorption and solubility, HEC showed intermediate values, and 3DP exhibited the highest values. (Fig. 4). These findings align with evidence associating the reduced hygroscopicity of milled PMMA with its dense network and lower porosity [5, 9]. For 3DP, increased sorption/solubility may be explained by microvoids at interlayer boundaries, a lower degree of double-bond conversion, and the use of more hydrophilic monomers typically present in additively manufactured photopolymers [6, 23]. Nevertheless, the limited disclosure of resin composition by manufacturers constrains more detailed mechanistic interpretation [24].

In addition to the manufacturing approach, differences in chemical composition likely contributed to the behavior observed among the evaluated material systems. CAD-M consisted predominantly of PMMA with EDMA and other additives and was supplied as an industrially polymerized block, whereas HEC was produced from a conventional powder–liquid system containing PMMA, methyl methacrylate, and a cross-linking agent. In contrast, the 3DP material was described by the manufacturer as a photopolymer formulation containing monomers, oligomers, photoinitiator, pigments, and stabilizers. These compositional differences may influence polymer network density, degree of conversion, molecular mobility, water affinity, and the amount of potentially leachable components, thereby affecting thermal behavior, sorption, solubility, and color stability. However, because the exact chemical formulation of the 3DP resin is not fully disclosed by the manufacturer, the contribution of individual components cannot be determined from the present study. Therefore, the observed differences should be interpreted as resulting from the combined influence of material composition and processing characteristics rather than from the manufacturing route alone [9, 10, 15, 23, 25].

Color stability also differed among the evaluated material systems. The 3DP group showed significantly higher ΔE00 values than both HEC and CAD-M at 3 and 7 days, whereas HEC and CAD-M did not differ significantly from each other at either time point. The greater discoloration observed for 3DP may be related to its lower Tg and greater molecular mobility, which may favor water uptake and the penetration of staining molecules. In this sense, color instability may be influenced not only by surface characteristics but also by the intrinsic thermal and molecular characteristics of the polymer network.

Surface analyses provided complementary information regarding the morphological characteristics of the evaluated material systems. Representative AFM images indicated qualitative differences in nanoscale surface appearance among the materials (Fig. 2). The 3DP samples exhibited a more irregular surface appearance, whereas CAD-M showed a comparatively more uniform topography and HEC displayed distinct morphological features. Although matched 15 × 15 μm scan areas were used, the AFM analysis was qualitative and height scales differed among materials. Therefore, no quantitative comparison of surface roughness or direct association between nanoscale topography and color change can be established from the present data.

SEM micrographs further illustrated morphological differences among the material systems (Fig. 2). The 3DP surfaces exhibited layer boundaries, discontinuities, and depressions characteristic of additive manufacturing, whereas CAD-M showed machining-related linear features with minimal evidence of surface defects. HEC exhibited interglobular features and occasional microvoids consistent with powder–liquid processing. These observations should be interpreted descriptively, as the present surface analyses were not designed to establish quantitative associations between morphology and the thermal, hygroscopic, or color outcomes.

The residual monomer content is also relevant to hygroscopic behavior and dimensional stability, as lower conversion can promote higher sorption, swelling, and leaching of soluble components [10]. This underscores the importance of optimizing postcuring protocols for 3DP materials. Recent evidence indicates that extended postpolymerization can increase the conversion and improve the mechanical properties of additively manufactured resins, although it may not fully overcome the vulnerabilities imposed by interlayer interfaces [25].

From a clinical perspective, although 3DP manufacturing offers important advantages in workflow efficiency and digital customization, the present results showed lower thermal stability and greater hygroscopicity and discoloration for the specific 3DP material tested. CAD-M showed the lowest water sorption and solubility, whereas CAD-M and HEC exhibited comparable thermal-viscoelastic behavior. Future clinical trials, such as those proposed by Rabel et al. [26], remain essential to determine whether these laboratory differences translate into clinically meaningful differences in wear behavior, comfort, and splint longevity in vivo.

This study has limitations that should be considered when interpreting the findings. Only one commercial material was evaluated for each manufacturing approach, and the tested materials differed not only in processing method but also in chemical composition. Therefore, the observed differences cannot be attributed exclusively to the manufacturing route. In addition, the AFM analysis was qualitative and performed after the color-change protocol, and quantitative surface roughness measurements were not obtained. The aging conditions were limited to water storage and coffee immersion and did not reproduce mechanical fatigue, pH fluctuations, or the complexity of the intraoral environment. Therefore, the present findings should be interpreted as a laboratory comparison of the specific material systems investigated. Future studies including multiple commercial materials within each manufacturing category and clinically relevant aging protocols are needed to confirm whether these differences persist under intraoral conditions.

## Conclusions

The three evaluated commercial occlusal splint material systems showed significant differences in thermal-viscoelastic behavior, hygroscopic properties, and color stability, while qualitative surface analyses revealed distinct morphological characteristics. CAD-M and HEC exhibited comparable tan δ, glass transition temperature, and thermal heterogeneity, whereas 3DP showed lower tan δ and Tg and greater thermal heterogeneity than both PMMA-based systems. CAD-M presented the lowest water sorption and solubility, while 3DP showed the highest values for these properties and significantly greater discoloration than CAD-M and HEC. Qualitative surface analyses also indicated distinct post-immersion morphological patterns among the materials, with 3DP exhibiting a more irregular surface appearance. Because each manufacturing approach was represented by a single commercial material with a distinct composition, the observed differences cannot be attributed exclusively to the manufacturing route. Therefore, the findings should be interpreted as a comparison of the specific material systems investigated. Further studies including multiple commercial materials within each manufacturing category, quantitative surface analyses, clinically relevant aging protocols, and clinical validation are needed to determine whether these laboratory differences translate into differences in long-term clinical performance.

## Supplementary Material

Below is the link to the electronic supplementary material.

Supplementary Material

## Data Availability

No datasets were generated or analysed during the current study.

## References

[1] De Laat A, Stappaerts K, Papy S (2003) Counseling and physical therapy as treatment for myofascial pain of the masticatory system. J Orofac Pain 17(1):42–49 12756930

[2] Fricton J, Look JO, Wright E, Alencar FG Jr, Chen H, Lang M, Ouyang W, Velly AM (2010) Systematic review and meta-analysis of randomized controlled trials evaluating intraoral orthopedic appliances for temporomandibular disorders. J Orofac Pain 24(3):237–254 20664825

[3] Proff P, Fanghänel J, Gedrange T, Kordaß B (2007) A Michigan-type occlusal splint with spring-loaded mandibular protrusion functionality for treatment of anterior disk dislocation with reduction. Ann Anat 189(4):362–366 10.1016/j.aanat.2007.02.026 17695993

[4] Gil-Martínez A, Paris-Alemany A, López-de-Uralde-Villanueva I et al (2018) Management of pain in patients with temporomandibular disorder (TMD): challenges and solutions. J Pain Res 11:571–587 10.2147/JPR.S127950 PMC5859913 29588615

[5] Benli M, Ural C, Simsek B, Yildirim C (2020) Surface roughness and wear behavior of occlusal splint materials made of contemporary and high-performance polymers. Odontology 108:240–250 10.1007/s10266-019-00463-1 31612354

[6] Väyrynen VOE, Tanner J, Vallittu PK (2016) The anisotropicity of the flexural properties of an occlusal device material processed by stereolithography. J Prosthet Dent 116(5):811–817 10.1016/j.prosdent.2016.03.018 27312654

[7] Kurt H, Özcan M, Arikan A, Ramoglu S (2012) Two-body wear of occlusal splint materials. J Oral Rehabil 39(8):584–590 10.1111/j.1365-2842.2012.02301.x 22486490

[8] Nekora-Azak A, Evlioglu G, Alptekin K, Cakarer S (2009) Patient responses to vacuum-formed splints compared to heat-cured acrylic splints: pilot study. J Maxillofac Oral Surg 8:31–33 10.1007/s12663-009-0008-9 PMC3454027 23139466

[9] Berli C, Thieringer FM, Sharma N, Sharma A, Müller JA (2020) Comparison of the mechanical properties of pressed, milled, and 3D-printed resins for occlusal devices. J Prosthet Dent 124(6):780–786 10.1016/j.prosdent.2019.10.024 31955837

[10] Perea-Lowery L, Gibreel M, Vallittu PK (2021) Evaluation of the mechanical properties and degree of conversion of 3D-printed splint material. J Mech Behav Biomed Mater 115:104254 10.1016/j.jmbbm.2020.104254 33333480

[11] Venezia P, Torsello F, Cavalcanti R, Ortolani M, Cicciù M (2019) Digital manufacturing of occlusal splint: from intraoral scanning to 3D printing. J Osseointegration 11(4):535–539

[12] Edelhoff D, Beuer F, Güth JF (2017) CAD/CAM splints for the functional and esthetic evaluation of newly defined occlusal dimensions. Quintessence Int 48(3):181–184 10.3290/j.qi.a37641 28232961

[13] Wang SM, Han L, Bai W et al (2019) Preliminary clinical application of a complete digital workflow for designing and manufacturing occlusal splints for sleep bruxism. Beijing Da Xue Xue Bao Yi Xue Ban 51(1):105–110 10.19723/j.issn.1671-167X.2019.01.019 PMC7433544 30773553

[14] Ryu JE, Park JM, Jeong CM, Huh JB (2020) Marginal and internal fit of 3D-printed provisional crowns according to build directions. J Adv Prosthodont 12(4):225–232 10.4047/jap.2020.12.4.225 PMC7449822 32879713

[15] Finck NS, Fraga MAA, Correr AB, Dalmaschio CJ, Rodrigues CS, Moraes RR (2024) Effects of solvent type and UV post-cure time on 3D-printed restorative polymers. Dent Mater 40(3):451–457. 10.1016/j.dental.2024.01.009 38129193

[16] ASTM International (2023) ASTM E1640-18: Standard test method for assignment of the glass transition temperature by dynamic mechanical analysis. ASTM International, West Conshohocken, PA

[17] ASTM International (2024) ASTM D7028-07: Standard test method for glass transition temperature (DMA Tg) of polymer matrix composites by dynamic mechanical analysis (DMA). ASTM International, West Conshohocken, PA

[18] International Organization for Standardization (2019) ISO 4049:2019 Dentistry—polymer-based restorative materials. International Organization for Standardization, Geneva

[19] Valenti C, Correr AB, Moraes RR et al (2024) Mechanical and biological properties of polymer materials for oral appliances produced with additive 3D printing and subtractive CAD-CAM techniques compared to conventional methods: a systematic review and meta-analysis. Clin Oral Investig 28:396 10.1007/s00784-024-05772-6 38916682

[20] Carvalho-Silva JM, Marinho VT, Lopez VP, Teixeira ABV, Ferreira I, de Campos MR, Dos Reis AC (2025) Printed versus heat-polymerized resin for occlusal devices: evaluation of physical and mechanical properties after brushing and microbial adhesion. J Prosthodont. 10.1111/jopr.14012 39763160

[21] Abad-Coronel C, Moraes RR, Correr AB et al (2023) Comparative analysis between conventional acrylic, CAD/CAM-milled, and 3D CAD/CAM-printed occlusal splints. Materials 16(18):6269 10.3390/ma16186269 PMC10532716 37763547

[22] Kageyama T (2023) Are 3D-printed resin surgical splints safe to use in the mouth? A comparative in vitro study. Ann 3D Print Med 9:100097

[23] Maleki T, Gibreel M, Vallittu PK (2024) Mechanical and physical properties of splint materials for oral appliances produced by additive, subtractive, and conventional manufacturing. Dent Mater 40(8):1171–1183 10.1016/j.dental.2024.05.030 38851965

[24] Gibreel M, Perea-Lowery L, Vallittu PK (2022) Two-body wear and surface hardness of occlusal splint materials. Dent Mater J 41(6):916–922 10.4012/dmj.2022-100 36288940

[25] De Santis LR, Correr AB, Moraes RR (2025) Aging and post-polymerization effects on conversion degree and properties of additive splint materials. Braz Oral Res 39:e017 10.1590/1807-3107bor-2025.vol39.017 PMC11808695 39936716

[26] Rabel K, Elsholk S, Nold J et al (2024) 3D-printed versus milled stabilization splints for the management of bruxism and temporomandibular disorders: study protocol for a randomized prospective single-blinded crossover trial. Trials 25(1):589 10.1186/s13063-024-08437-7 PMC11376033 39238023

