# Supplementary material for: Comparison of three occlusal splint material systems manufactured by conventional, subtractive, and additive techniques
Source: Clin Oral Investig. 2026 Nov 9;30(12):562. doi: 10.1007/s00784-026-07250-7 (PMC13745416; doi:10.1007/s00784-026-07250-7)
Supplement: Supplementary file 1 [file 784_2026_7250_MOESM1_ESM.docx]

 Supplementary Fig. S1. Individual dynamic mechanical analysis curves obtained at 10 Hz for the evaluated material systems. (A) Storage modulus (E′), (B) loss modulus (E″), and (C) tan δ as a function of temperature for the five specimens of CAD-M, HEC, and 3DP. (D) Comparison of the first and second heating tan δ curves for 3DP, illustrating the shift in the tan δ peak between heating cycles.
